# Editorial: New insights into yoga and mental health

**DOI:** 10.3389/fnhum.2023.1239411

**Published:** 2023-06-30

**Authors:** Vijaya Majumdar, N. K. Manjunath

**Affiliations:** Division of Life Sciences, Swami Vivekananda Yoga Anusandhana Sasmsthana, Bengaluru, India

**Keywords:** yoga, mental health, randomized controlled trial (RCT), intervention, updates

Mental disorders are increasingly recognized as leading contributors to the global disease burden, and of the years lived with disability (Patel et al., 2016, 2018). The COVID-19 pandemic has further fueled this global mental health crisis (Vindegaard and Benros, 2020; Radfar et al., 2021). The universal coverage of cost-effective interventions for mental disorders is a crucial strategy to tackle the rise of mental health problems (Chisholm et al., 2012; Patel et al., 2016). Given its benefits on somatic and mental health factors, yoga could be a good fit as a cost-effective treatment for mental disorders. This notion was pragmatically implemented during the COVID-19 pandemic, as reflected in the substantial rise in the interest toward adopting healthier lifestyle choices (Jindal et al., 2021). Numerous empirical studies have confirmed the beneficial effects of yoga practice on various mental health indicators (Gothe et al., 2019). As a combination of physical postures, rhythmic breathing, and meditative exercise, yoga provides a unique holistic mind-body experience. The mental health-related evidence on yoga also encompasses its positive effects on brain structure and function, supported by neuroimaging data (Gothe et al., 2019). A systematic review by Larson-Meyer (2016), reported that while some specific yoga postures could fall under the category of metabolically exerting (with energy expenditures >3 METS), most of the other practices align with the American College of Sports Medicine's criteria of “light-intensity physical activity.”

This Research Topic “New Insights into Yoga and Mental Health” aims to provide an update on the critical developments in yoga-based interventional research for mental health. Overall, the compendium included four articles about Yoga and Mental Health.

The Research Topic includes two interesting randomized controlled trials (RCTs). The article by Haller et al. tested the efficacy of pranayama, meditative yoga breathing and breath holding as complimentary stabilization techniques for Trauma-Focused Cognitive Behavioral Therapy in patients with post-traumatic stress disorder (PTSD). TF-CBT is the first line of treatment for patients with PTSD and uses stabilization techniques before the confrontation. The authors reported that in patients without concurrent somatoform disorders, integrating pranayama into TF-CBT might reduce their post-traumatic symptoms and increase their mental quality of life more efficiently than TF-CBT alone. However, the authors also emphasized the preliminary nature of the findings and the need to replicate the results by intention-to-treat analyses. These findings are relevant given the reported flare-up of post-traumatic stress symptoms (96.2%) as mental health consequences of COVID-19 (Vindegaard and Benros, 2020).

Another clinical trial of this Research Topic by Shidhaye et al., addressed maternal mental health outcomes (perceived stress, quality of life) in pregnant women from rural settings of India, representing a marginalized sector of the population with enhanced vulnerability during the pandemic (Busch-Hallen et al., 2020). Yoga-based intervention to improve maternal Mental health and iMmunity during the COVID-19 crisis (Yoga-M2 trial) was a single-blind individual randomized parallel group-controlled pilot trial with a 1:1 allocation ratio implemented to address this gap. The pilot trial was unique in being the first to test the efficacy and feasibility of a prenatal yoga-based intervention to improve women's maternal wellbeing in rural India's deprived settings amidst the COVID-19 crisis. Positively, the authors were able to recruit pregnant women within 9 weeks and retain almost all of them until the end of the trial. Though the predefined outcomes were not statistically significant, there were subjective beneficial outcomes reported by the women, which the participants accounted for their adherence.

In the study protocol by Inbaraj et al., the authors presented the design and method of a randomized clinical trial, wherein they aimed to study the Impact of integrated yoga therapy on cognitive impairment and cardiac dysfunction regarding the quality of life in breast cancer patients undergoing chemotherapy. These subtle adverse effects, particularly changes in memory or cognition, remain unattended in many women, affecting their memory, and the ability to think clearly during and after chemotherapy (Vitali et al., 2017). The trial would also include assessments of changes in the neural circuitry using functional magnetic resonance imaging (fMRI). Overall the study might be an imperative aid in establishing the prophylactic potential of yoga for breast cancer patients. The results of this study could also be extended to chemotherapy-exposed individuals with other solid cancer types with cognitive and cardiac issues.

This research compendium also included an interesting state-of-the-art narrative review article on the coexisting condition of irritable bowel syndrome and fibromyalgia. These two health conditions share common pathophysiological mechanisms; sensitization of peripheral and central pain pathways and autonomic dysfunction. The conjuncture of these two conditions led to increased symptom severity, mental health comorbidities, and decreased quality of life. On an individual basis, mind-body interventions have been reported to benefit both the conditions and influence central pain syndromes and autonomic dysregulation. The authors evaluated the current evidence on the role of mind-body therapies in the coexisting state. They reported the limited availability of data in co-diagnosed patients. They also warranted the need for high-quality trials to tailor the programs individually.

Given the wide diversity in psychological morbidities and scarcity of data, it becomes imperative to conduct studies to address the gaps in the area, ranging from epidemiology to intervention research.

## Author contributions

VM: manuscript writing. NM: review of manuscript. All authors contributed to the article and approved the submitted version.
